# Long-term impact of the SARS-CoV-2 pandemic on respiratory viruses in Germany

**DOI:** 10.1186/s12889-025-23983-8

**Published:** 2025-08-05

**Authors:** Ralf Eggeling, Florian König, Lisa Koeppel, Laura-Inés Böhler, Michael Böhm, Norbert Schmeißer, Nico Pfeifer, Rolf Kaiser, Clinical Virology Network

**Affiliations:** 1 Department of Computer Science, University of Tübingen, Tübingen, Germany; 2 Department of Infectious Disease and Tropical Medicine, Heidelberg University Hospital, Heidelberg, Germany; 3 Institute of Virology, Faculty of Medicine and University Hospital Cologne, Köln, Germany; 4 Medeora GmbH, Köln, Germany; 5 Institute for Bioinformatics and Medical Informatics (IBMI), University of Tübingen, Tübingen, Germany

**Keywords:** Respiratory viruses, Seasonality, Non-pharmaceutical interventions, SARS-CoV-2 pandemic, COVID-19, Coinfections

## Abstract

**Background:**

Respiratory viral diseases are one of the greatest challenges facing our healthcare system, with them being one of the main causes of death. This has been demonstrated once again by the impact of the SARS-CoV-2 pandemic in recent years. We study the impact of the SARS-CoV-2 pandemic on the prevalence of respiratory viruses by analysing a subset of the Clinical Virology network database, covering 2,216,198 samples tested for 18 different viral pathogens in the time span from 2010 to 2024.

**Methods:**

We calculated the prevalence of 17 respiratory viruses before and after onset of the SARS-CoV-2 pandemic and compared the degree of seasonality shift with a newly developed a metric dubbed seasonal disruption index. In addition, we compared coinfection statistics prior to and after the pandemic onset, and also studied the correlation of infection counts with non-pharmaceutical interventions in the time frame from early 2020 to end of 2022.

**Results:**

We found that the viral pathogens show a varying degree of seasonality disruption. It is largest among those that are known to show a highly seasonal behavior, namely Influenza and RSV, the latter having the highest seasonal disruption index. Most perennial viruses continued to appear throughout the year. Coinfections occurred before and after the pandemic; patterns before and after pandemic onset are surprisingly similar. The occurrence of most viruses is nonlinearly correlated with the degree of non-pharmaceutical interventions.

**Conclusion:**

The SARS-CoV-2 pandemic had a considerable impact on the occurrence and seasonality of other respiratory viruses. While nearly all seasonality patterns were initially disrupted due to the heavy non-pharmaceutical interventions, viruses are regaining their pre-pandemic seasonality.

**Supplementary Information:**

The online version contains supplementary material available at 10.1186/s12889-025-23983-8.

## Background

Respiratory viral illnesses are one of the key challenges facing our healthcare systems. Most prominently perceived in the population, each year, an estimated 5% to 15% of all people are affected by respiratory tract infections caused by influenza viruses alone [1]. While some outbreaks such as MERS (Middle East respiratory syndrome coronavirus) in 2012 or SARS (severe acute respiratory syndrome) in 2003 remained fairly localized with a limited number of casualties, others such as the Spanish flu (1918-1920) and, more recently, the SARS-CoV-2 pandemic, had a profound impact on human development throughout the world [2–4]. In response, considerable efforts are taken to prepare for future pandemics and to limit their societal and economic burdens [5, 6].

One aspect of preparedness is the continuous observation of known respiratory virus dynamics to enable quick responses to local and global outbreaks to protect at-risk groups [7, 8]. Prior long-term studies revealed predictable seasonality patterns for such viral diseases [9–11], for example, enabling preparations of the new influenza vaccines [7, 12]. It is also important to assess virus-virus interactions, both synergistic and antagonistic, as co-infected patients might suffer from worse health outcomes [2, 10, 13].

Several studies suggest that previously identified long-term seasonalities before the pandemic have been distorted, leading to missing seasons or shifts in seasonalities during and after the pandemic [14–16]. Such investigations into the altered behavior of respiratory viral strains have been made in several countries [17, 18], including Germany [14, 19–21]. An extensively studied example is the respiratory syncytial virus (RSV), with multiple studies observing an entire missing season [15, 22, 23]. This has been correlated with non-pharmaceuticcal interventions (NPIs), implemented to prevent the SARS-CoV-2 pandemic completely overloading national healthcare systems. The reduction in transmissibility hoped to achieve for SARS-CoV-2 also affected the transmission of other previously analyzed respiratory pathogens [15–17, 19, 23, 24]. There are also a few studies regarding coinfections between SARS-CoV-2 and other pathogens [25, 26].

Although many insights regarding the impact of the SARS-CoV-2 pandemic on infection dynamics have been gained, there are still gaps in the literature. Most studies only consider a sub-population [14, 19] or small region [17, 20] rather than considering the entire population of a country due to a lack of data on a large scale. Many studies are also limited by considering only one [16, 23, 24] or a small number [17, 18, 20, 22] of viruses, and thus also lack or do not even attempt a systematic comparison. Furthermore, many studies were published relatively early in the pandemic [14, 15, 17, 18, 21, 22]. Hence, they only focus on a short period compared to the previously identified dynamic patterns and do not permit to assess whether any seasonal shifts and newly identified patterns will remain stable or eventually return to the state prior to the pandemic. Finally, coinfection studies in the literature often involve SARS-CoV-2 and influenza [25], widely neglecting interactions among other viruses.

In this article, we attempt to fill some of the gaps by addressing the aforementioned limitations. We build upon our prior study on the data from the clinical-virology.net [9] in order to cover a large number of contributing sites in Germany and infection counts for 18 different viruses. These data span the period before, during, and after the pandemic, up until April 2024. As a result, four full years after the start of the pandemic are available for a comparative analysis against the prepandemic timeframe. In order to quantify seasonal shifts and other disruptions of viral dynamics during the pandemic and, most importantly, compare the behavior of different viruses, we propose a *seasonality disruption index* (SDI). We further study the correlation of infection counts with NPIs, also with the primary aim of comparing viruses. In addition, we study possible changes in coinfection patterns among all pairs of viruses before and after the start of the pandemic.

## Methods

This section contains a description of the data, followed by a general outline of our analysis methodology, and a description of our newly developed method for the quantification of seasonality disruption.

### Data collection and preprocessing

Since November 2009, the clinical-virology.net, formerly known as RespVir network, has collected multiplex test records for 17 virus infections from patients who showed respiratory infection symptoms. Since January 2020, SARS-CoV-2 has been included in the list of tested viruses. The records stem from up to 47 sites, although the tested viruses and covered time spans differ greatly across sites. We used the clinical virology network (CVN) database updated until April 2024, using the data from January 2010 onwards to be consistent with our previous work [9], and carried out the following data cleaning and preprocessing steps.

First we removed all biologically implausible data entries. There are 5,201 (of 2,222,843) data points that showed positive results against more than four viruses. Additionally, one site had 1444 positive HBoV test results in early 2018 (January–April), with a nearly 100% positivity rate. Both cases are likely caused by incorrect data entry, for instance by swapping positive and negative labels, so we excluded the affected data points entirely. Furthermore one site marked each of 211 positive test results for Influenza A subtype H1N1 also as positive for subtype H3N2. We excluded these counts for the coinfection analysis specifically.

Second, the tests used by some sites do not differentiate between certain virus types or subtypes, such as FLUA-generic, which cannot distinguish between influenza A H1N1 and H3N2. In this study, we excluded these combitests and retained only the 18 tests against single virus types. This eliminates 6,771 data points that comprise only combitests.

Third, we filtered out 97,710 data points from non-German test sites, as the one objective of the project was to correlate infection counts with non-pharmaceutical interventions, which needs to be done on the country level. For all countries aside from Germany, samples sizes in the CVN database are too small to draw statistically significant conclusions.

An overview and a quantitative description of the final data set after preprocessing, that is all virus names, abbreviations, absolute and relative infection counts, is included in the supplementary material. Unsurprisingly, the number of tests and positive results for SARS-CoV-2 dwarfs those of the remaining viruses, amounting for more than all others combined. But also the percentage of positive tests results is with more than 24% considerably higher than for all other viruses, where positive percentages range between 1% and 16%.

### Analysis methodology

In our analysis, we first inspected the absolute infection and relative infection counts for each virus aside from SARS-CoV-2 in a time-series fashion for the period of January 2010 to April 2024. Relative counts are defined as the absolute number of positive test results divided by the number of tests conducted.

Subsequently, we investigated how the pandemic affected virus dynamics and analysed alterations in seasonal outbreak behaviour. For this purpose, we developed a statistical measure, the so-called *disruption statistics*, to quantify the disruption of seasonal patterns. It states the difference between the observed infections and the expected infections based on the median from the past ten years. More details and a mathematical description is provided in the following Quantification of seasonality disruption section. Using the disruption statistics and identical parametrization, we identified disruption profiles, further enabling a clustering of the viruses according to their profile in a dendogram. All of the aforementioned measures allowed an objective comparison of seasonal disruption across viruses.

We further studied the association of infection counts and NPIs. For this purpose, we included SARS-CoV-2 and quantified NPIs using the Stringency Index from the Oxford Covid-19 Government Response Tracker (OxCGRT) [27]. This index combines several factors such as school closings and restrictions on public gatherings. Since the stringency index is available only for the time period January 2020 – December 2022, all analyses regarding NPIs are limited to that time period. Our analysis was performed on a monthly aggregation which is why we averaged the daily stringency index values for each month to suit our format.

Lastly, we investigated coinfections among all 18 viruses before and after the start of the pandemic. Due to the small sample sizes post 2020, it was not possible to repeat the analysis as in a previous study [9], where we analyzed virus pairs with a score that shows statistically significant increase or decrease in coinfections compared to what would be expected by chance. Instead in the present study we solely compared coinfection counts before and after the start of the pandemic.

### Quantification of seasonality disruption

To examine how the pandemic affected virus dynamics and seasonal outbreak behavior, we define statistical measures to quantify the disruption of seasonal virus patterns in the following. For an arbitrary virus, let $n_{\text {id}(y,m)}$ denote the absolute number of infections in month *m* of year *y*, whereby the subscript

$$\begin{aligned} \text {id}(y,m)=12*(y-2010)+m \end{aligned}$$

returns the global index of the month in the time period under consideration, i.e., how many months have passed since the beginning of 2010. We calculate smoothed infection counts with a window size of *w* as

$$\begin{aligned} \tilde{n}_{i} = \frac{1}{2w+1}\sum \limits _{j=i-w}^{i+w} n_{j} \end{aligned}$$

for $i>m$. Let *d* denote the number of past seasons we wish to consider and $\alpha$ denote a small pseudocount to avoid infinity in cases of zero infections in a particular month. We define the *disruption statistics*

$$\begin{aligned} \text {DS}_{i} = \text {log2}\left( \frac{\tilde{n}_{i}+\alpha }{\text {median}(\tilde{n}_{i-12d},\dots ,\tilde{n}_{i-12})+\alpha }\right) \end{aligned}$$

This statistics quantifies how much more/less frequent the infection counts in month *i* were compared to an expected value, which is based on the median of the past *d* seasons as the baseline. If not explicitly specified otherwise, we use $w=2$, $\alpha =1$, and $d=10$ for all studies.

Let us consider a time period between months *a* and *b* (with $b>a$). We call the vector of disruption statistics values in the chosen time period *disruption profile*. The *seasonality disruption index* (SDI) quantifies the total disruption of infection seasonality between months *a* and *b* by

$$\begin{aligned} \text {SDI}(a,b) = \sqrt{\frac{1}{b-a}\sum \limits _{i=a}^{b} \text {DS}_{i}^2} \end{aligned}$$

which is essentially a root mean squared deviation of the disruption profile to a straight line at zero (no seasonality disruption). The key advantage of this metric over previously used Kullback-Leibler divergence based quantification of seasonality [9] is that it allows quantification of seasonality disruption for arbitrary time spans and not only whole years.

Further, we considered the disruption statistics for each virus in the given time period, e.g., $(\text {DS}_{a},\dots ,\text {DS}_{b})$ as feature vector and applied agglomerative clustering with average linkage using the feature vectors of all viruses as data points and squared Euclidean distance.

## Results

This Result section first describe infection trajectories over time for a few selected viruses, motivating the need for a unified quantitative evaluation, followed by large-scale analyses for all viruses regarding seasonality disruption, correlation with non-pharmaceutical interventions, and coinfections.

### Infection counts over time

**Fig. 1:**
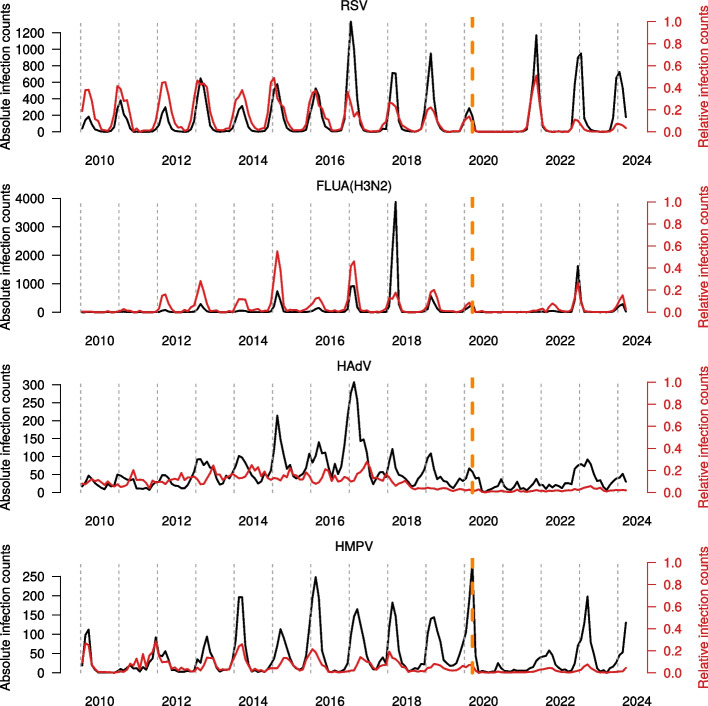
Positive tests for RSV, Influenza A H3N2 (FLUA(H3N2)), HAdV, and HMPV aggregated by month in the time period January 2010 until March 2024. The red line displays relative counts, i.e., positive tests divided by the number of tests. The vertical dashed orange line marks the start of the SARS-CoV-2 pandemic. See supplement for other viruses

We first inspected the absolute infection and relative infection counts for each virus in the time period January 2010 to April 2024. Figure 1 displays four typical examples, identical plots for the remaining viruses are shown in the supplementary material. Despite only using German data here, all plots are consistent up to 2019 with the previous analysis of the CVN data [9]. The extended time period also gives insights on the impact of the SARS-CoV-2 pandemic on the infection dynamics.

As a first example, we use the human respiratory syncytial virus (RSV), which can cause severe infections in premature newborn, young children, elderly and immunocompromised [28, 29]. These groups are candidates for either passive immunisation (children) for vaccination (elderly, immunocompromised).

We observed a clear seasonal occurrence pattern with annual peaks in January and February, being disrupted at the onset of the pandemic (Fig. 1) [15, 16]. A peak in early 2021, expected based on the typical seasonal pattern in the years before, is missing. The following peak emerged in fall 2021 and the peaks of the subsequent two seasons appeared in December instead of January/February. The RSV occurrence pattern recovered from the pandemic disruption with respect to frequency, however, the seasonal peak is still shifted by two months.

Prior to the pandemic, the seasonality pattern of influenza A subtype H3N2 remained consistent over the years regarding onset and duration. Yet, the size of the individual waves differed. Upon the pandemic influenza A H3N2 could not be observed for nearly two seasons before re-appearing at the end of 2022 in a uni-modal wave. Until the end of the study period, no return to the prepandemic seasonal patterns could be seen.

Prepandemically, the human adenovirus (HAdV) was known to belong to the perennial group showing little preference for a particular season but rather occurred in a fluctuating fashion throughout the year. In contrast to other viruses, almost no disruption of absolute infection numbers could be observed upon the SARS-CoV-2 pandemic. The relative HAdV frequency (regarding number of tests) decreased in recent years. Anyhow, this trend has started before the pandemic.

Human Metapneumovirus (HMPV) is a respiratory pathogen, phylogenetically related to RSV and Parainfluenza 1-4, which is not as intensively observed as Influenza, SARS-CoV-2 or RSV. It is a severe burden not only in children but also in adults, especially those over 65 years of age [30–34]. Infections with HMPV are detected and reported within our respiratory pathogens network, CVN, since the beginning of our activity [9]. More recently, vaccines have been developed and have now entered clinical studies [35, 36]. Here, we describe the re-emergence of HMPV after the pandemic.

Prior to the pandemic, HMPV showed infection peaks in winter (December–January), although there is at least one season (2011) in which low infection counts were detected and therefore a typical peak can hardly be identified. There is a notable peak in absolute infection counts located at the pandemic onset in early 2020 with an absence in of infections in 2021. The sparsity and sporadic occurrence after the start of the pandemic are in line with prepandemic patterns and may be explained by natural variation inherent to the virus. Notably, relative infection counts remained remarkably low after the start of the pandemic.

### Comparison of seasonality disruption

Prepandemic seasonality trends differed to a large degree among the considered viruses as described in our previous work [9]. In order to obtain a quantitative measure for the disruption of the seasonality patterns, we applied the seasonality disruption index (Quantification of seasonality disruption section). We exemplified the index by RSV, the detailed plots for the other viruses are shown in the supplementary material.

**Fig. 2:**
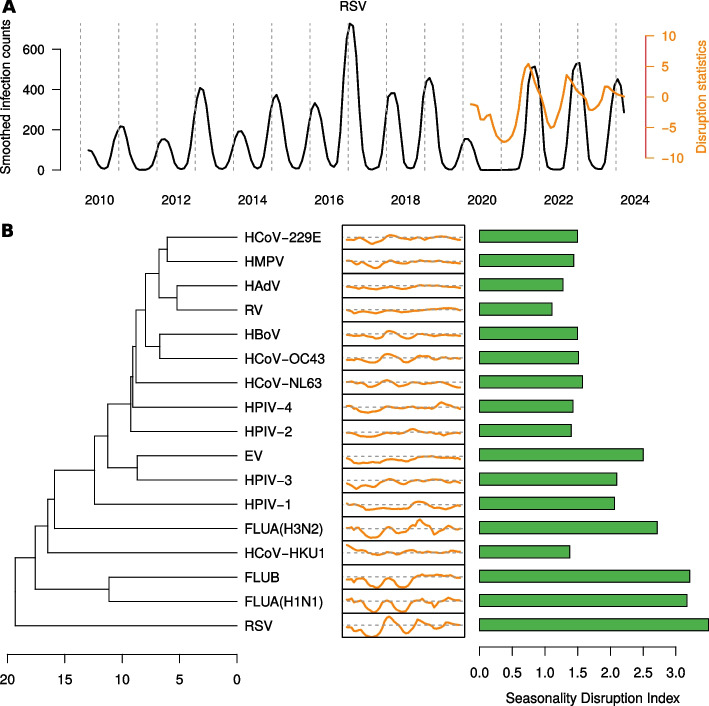
Quantifying seasonality disruptions. **A** Smoothed absolute infection counts for RSV in the time period 2010–2024 (black line). The orange line displays the disruption statistics, i.e. the difference between the observed infections and the expected infections based on the median from the past 10 years. **B** The 17 viruses are hierarchically clustered according to their disruption statistics profile. The middle plots show the disruption statistics from 2020-2024 in the range from -7 to 7, where the dashed line represents value zero. The barplot shows for each virus the aggregated disruption, i.e., the sum of the absolute disruption statistics over all months

Figure 2A displays smoothed absolute infection counts for the entire time period. It also contains the disruption profile after pandemic onset (March 2020 to March 2024). The disruption profile fluctuates in the range of -6 in early 2021 to +5 in late 2021. Thus, it aligns with the presence and absence of expected and unexpected peaks.

We used the disruption profiles as feature vectors and applied hierarchical clustering. The resulting dendrogram with each disruption profile plotted adjacent to the corresponding leaf in the tree is shown in Fig. 2B. Additionally, the plot also displays the seasonality disruption index (SDI) for each virus, which is the root mean square deviation of the disruption profile from a null vector.

Figure 2B reveals that RSV shows the highest SDI and is also least similar to any other virus in the selection in terms of disruption profile. RSV frequency was unexpectedly low upon the onset of the pandemic (Fig. 2A). Nevertheless, RSV occurrence strongly increased in late 2021 after a period of little detection. It should be noted that HPMV shows both a different original seasonal trend and a different disruption, despite being genetically closely related to RSV and causing similar symptoms [37].

While some viruses such as influenza A (FLUA(H3N2) and FLUA(H1N1)) also exhibit a fluctuating disruption profile and thus a relatively high SDI, others, such as the non-enveloped viruses (Rhinovirus(V), EV, HAdV) less little seasonal disruption, which might be explained by them being perennial [9], not exhibiting a strong seasonality to begin with. In general, viruses that exhibited a stronger seasonality pre-pandemically also have a higher SDI, but the correlation is not perfect: Human parainfluenza 3 (HPIV-3), for instance, shows a relatively high SDI simply due to the absence of infection counts early in the pandemic. The dendrogram resulting from clustering disruption profiles resembles the clustering according to the pre-pandemic seasonal patterns [9], but not perfectly.

### Infection counts vs non-pharmaceutical interventions

**Fig. 3:**
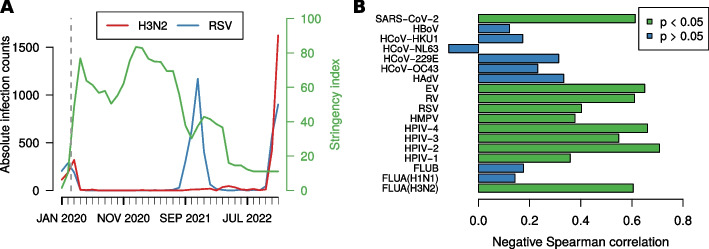
Infections and NPIs. **A** Overlay of infection counts and stringency index for influenza A H3N2 and RSV. **B** (Negative) Spearman correlation of stringency index and absolute infection counts for the time period from March 2020 to December 2022 for each virus under consideration. An equivalent plot additionally including January and February 2020 and Pearson correlation plots for both timeframes are shown in the supplement

Figure 3A displays an overlay of the stringency index and infection counts for the examples of RSV and FLUA(H3N2); similar plots for each individual virus under consideration are shown in the supplement.

For RSV, it is remarkable that after the pandemic onset in March 2020, the infection counts remain low as long as the stringency index is above a value of 60. Once restrictions were loosened (late 2021), the RSV wave appeared. A small increase in the stringency index then coincided with the end of aforementioned peak. Finally, in late 2022, when all NPI related public health measures aside from public information campaigns were terminated, the infection counts for RSV rose once again. Influenza A(H3N2) disappeared with rising stringency index in early 2020 and reappeared at the end of 2022. Unlike RSV, influenza A(H3N2) did not re-emerge within the pandemic period.

We calculated the Spearman (rank) correlation between the stringency index and infection counts for each virus and noted the corresponding *p*-values. The results in Fig. 3B show that many viruses, such as the RSV and FLUA(H3N2), exhibit a moderate yet significant negative correlation between infection counts and NPIs. However, this is not true for all considered viruses. Most notably, influenza B and A H1N1 have correlations of nearly zero, which is due to very small absolute infection numbers in the considered time period. As the SARS-CoV-2 pandemic reached Germany in March 2020 the first two months of 2020 were excluded for this analysis. If they were taken into account as well, correlations would be higher (Supplement). We also studied Pearson correlations among stringency index and infection counts (Supplement); here the resulting values are smaller in absolute numbers, which indicates that the relationship between NPIs and infection counts is to some degree nonlinear. We additionally repeated all correlation analyses with relative instead of absolute infection counts (Supplement). The results remain almost identical.

We also studied the relationship between the disruption statistics and NPIs (Supplement); here the correlations are even stronger, and there is little difference between Pearson and Spearman correlation.

### Coinfections

**Fig. 4:**
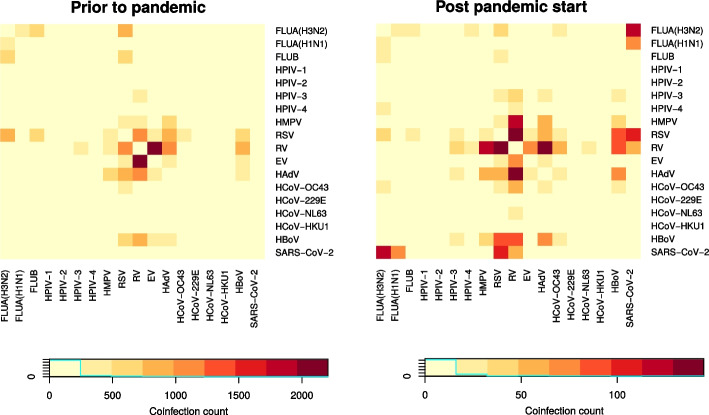
Heatmaps of coinfection counts prior to the pandemic (January 2010 – February 2020) and post pandemic start (March 2020 – April 2024)

We inspected coinfections among viruses for the provided study period and compared coinfection counts before (January 2010 to Feburary 2020) and after the start of the pandemic (March 2020 to April 2024) in a heatmap (Fig. 4). The general pattern appears fairly similar. Frequent coinfections, such as RSV and RV, persist. Most changes pertain human Bocavirus (HBoV) where coinfections became more frequent, in particular with RSV, HadV, and HMPV. Also HMPV/RV coinfections are occurring frequently. The only notable coinfections of SARS-CoV-2 reported in the CNV database are with RSV, RV, and both influenza A subtypes. The numbers are tiny in relation to the SARS-CoV-2 monoinfections, though (Supplement).

## Discussion

In this study, we performed a long-term analysis of respiratory viruses in Germany. We quantified seasonal shifts and compared the behaviour of 17 different viruses using self-developed seasonality disruption statistics.

All viruses showed a lower frequency during the pandemic. The reemergence of the viruses was different. We showed that for viruses that exhibit a specific seasonal outbreak pattern the pandemic had severe impact on the timing and the size of the outbreak waves. RSV constitutes a prime example as the disruption led to a missing wave in the 2020/2021 season followed by a reemergence in fall 2021. This could be interpreted either as a very delayed 2021 infection wave or an advanced 2022 wave. This is a unique behaviour among the considered viruses, further supported by its distinct disruption profile being least similar to any other virus. Therefore, it is plausible that it displays the highest SDI value.

Papenburg and Boivin [37] described different seasonal trends of RSV and HMPV, despite their genetic closeness and causing similar symptoms. Our study emphasizes this by not only showing different prepandemic seasonal trends but also various disruption statistics upon the COVID-19 pandemic onset. Furthermore, our results are in line with Terliesner et al.[14] who suggest that non-seasonal viruses (Rhinovirus/Enterovirus, Adenovirus) remained comparatively more stable upon the COVID-19 outbreak, while others showed out-of-season resurgences. The same holds true for the also closely phylogenetically related Parainfluenza viruses. RSV, HMPV and Parainfluenza belong to the family of Paramyxoviruses but clearly show a different biological behavior as demonstrated here.

Additionally, enveloped viruses such as RSV, FLUA(H3N2) and FLUA(H1N1) exhibit a fluctuating disruption profile and thus a relatively high SDI, whereas non-enveloped viruses, e.g. Rhinovirus or Adenovirus, show none to less seasonal behaviour and thus they show relatively little seasonal disruption. This is in line with Oh et al. [21], who suggests that the viral structure (enveloped versus non-enveloped viruses) might influence viruses dynamics during and after the SARS-CoV-2 prevention measures.

For Influenza A and B the disruption was also very prominent, as the disruption was even longer as compared to RSV and not directly related to the non-pharmaceutical-intervention index. There seems to be a relation as the more stringent the seasonality is, the longer the disruption period and the more difficult it is for the virus to get back into the pre-pandemic seasonality. Due to the vulnerability of the envelope of influenza viruses, they are very vulnerable to environmental influences. If this or other reasons like the reproduction number determine the differences in seasonality can only be speculated as we do not have such information collected in our network.

There is indeed a difference between the spreading of Influenza A H1N1, A H3N2 and the B variants (Yamagata and Victoria line). Different epidemiology between the different influenza variants is clearly visible and is verified by other networks like the Arbeitsgemeinschaft Influenza (AGI, www.RKI.de). Before the SARS-CoV-2 pandemic, different years sometimes had different epidemics showing different variants of influenza A and B. As we do not observe Influenza B Yamagata line derived virus strains after the pandemic, authorities discuss to exclude influenza B Yamagata line like virus vaccine from the recommended vaccine recommendation.

We were able to show statistically significant negative correlations between the case numbers and the implementation of non-pharmaceutical interventions for some viruses. The partially nonlinear correlation could be explained by an exponential increase in the infection counts and indeed Influenza A (H3N2), which has the largest difference between linear and rank correlation, has also the sharpest infection peak. Given this observation the nearly linear correlation between disruption statistics and NPIs is also plausible, as the former is defined on the logarithmic scale.

Aside from SARS-CoV-2, RSV is noted as the primary example for negative correlation between infection counts and NPIs. Yorsaeng et al. [18] could not identify a similar significant increase after implementation of NPIs as others have identified. However, not all viruses show this strong correlation. For some, such as Influenza B and Influenza A, subtype H1N1, it is due to virtually zero infections in the time period under consideration.

We analyzed a composite NPI index with disease incidence. Although individual NPIs, such as mask-wearing, social distancing, or travel restrictions, may vary in their impact, they are often implemented together. As a result, NPIs tend to be highly correlated and may exert synergistic or confounding effects, making it challenging to isolate the specific contribution of any single intervention [22]. Different studies aimed at disentangling these effects. Takeuchi et al. [24] used multiple regression analysis identifying associations between high mask use and high social distancing in 3 and 2 seasons with influenza. In contrast to the individual NPIs within the stringency index, they opted for more granular data for mask use and mobility data. Billard et al. [16] identified associations between school closures and stay-at-home orders in the RSV season using linear mixed models, though effect sizes were small.

We observed that coinfections occurred between different viruses and that this was relatively little affected by the pandemic. Most increases in coinfections are related to HBoV. It is discussed controversially whether HBoV is a mere bystander [38], a true pathogen in its own right [39] or coinfection with HBoV might lead to a more severe course of disease compared to single pathogen infections. We have no detailed clinical data in our network to either support clinical significance of HBOV or to prove the opposite, though. Other coinfection increases pertain RSV, which can be a consequence of the strong RSV wave in fall 2021.

Although our study is fairly comprehensive in terms of the long time period, number of viruses, and contributing hospitals, it has certain limitations. The heterogeneous nature of the CVN data may introduce biases as outlined in the following paragraphs.

Testing policies are not constant across time and location. As a consequence, neither absolute nor relative infection counts are free of bias. The former may overestimate infections and lead to false positive peaks in the infection dynamics with increased testing. Conversely, the latter may underestimate infections and lead to the missing of peaks in the infection dynamic in that situation. Since both forms of bias are possibly problematic, we display long-term trends for both statistics. We used absolute infection counts as basis for our main analysis, since our motivation was to study the possible absence of expected infection peaks post-pandemic onset, for which underestimation of infections is a greater concern than overestimation.

The analysed data set stemmed from the clinical virology network mainly comprising hospital data. Thus, the claims made in this study refers to this specific setting only. Tanislav et al. [40] analysed data from general practitioners and specialists and have shown a decrease in respiratory and gastrointestinal disease occurrence during the pandemic. However, no stratification by virus was performed. Further insight into the viral dynamics regarding surveillance detected by general practitioners would be interesting as a complementing comparison to the presented results. Similarly, there might be age-related differences in susceptibility or testing bias within the hospital setting.

A further limitation of our study include that the data are restricted to Germany. This may limit the generalizability of our findings. Viral dynamics and the effectiveness of public health measures differ significantly between countries due to variations in health care systems, political decisions, and societal behaviors. As such, our results may not be fully applicable to settings outside the German context. Moreover, pandemic response strategies, including testing regimes, mobility restrictions, and vaccination rollouts, varied widely across countries and over time. These differences make it difficult to draw direct comparisons or apply our findings to other regions without conducting localized analyses.

The disruption statistics and the seasonal disruption index are novel metrics introduced specifically for this study. One limitation is their dependence on the three hyperparameters *w*, *d*, and $\alpha$. Changing one or more of these values drastically will alter the results. While we consider the precise values used to provide a reasonable tradeoff between capturing as much information as possible while also eliminating noise on our CVN data, this does not necessarily generalize to other data sets. A systematic study of the effect of these parameters across different data sets could thus be a topic for future research.

## Conclusion

We provide a basis for data collection and a deeper understanding of virus dynamics and how non-pharmaceutical interventions affect the seasonality and occurrence of viruses is essential for preparing for upcoming seasons. Furthermore, this information is crucial for health policy, as it helps refine or develop new strategies to combat and predict seasonal peaks, as well as to improve diagnostics. Consequently, the challenges of new epidemics and even pandemics can be met.

## Supplementary Information

Supplementary Material 1.

## Data Availability

The clinical virology data this study is based on is available through a web interface at https://public.clinical-virology.net. The data export for this study is available on request from Rolf Kaiser. The code for running the studies on that export is available on request from Ralf Eggeling.
