# Supplementary material for: Long-term impact of the SARS-CoV-2 pandemic on respiratory viruses in Germany
Source: BMC Public Health. 2025 Aug 5;25:2654. doi: 10.1186/s12889-025-23983-8 (PMC12326739; doi:10.1186/s12889-025-23983-8)

Supplementary material to *Long-term impact of the SARS-CoV-2 pandemic on respiratory viruses in Germany*

Ralf Eggeling, Florian König, Lisa Koeppel, Laura-Inés Böhler, Michael Böhm, Norbert Schmeißer, Nico Pfeifer, Rolf Kaiser

1 Data set statistics

Neg. in % and Pos in % indiciate the counts divided by the number of tests for each virus, whereas Test prop. and Pos. Prop refers to the proportions of tests and positive results across the whole set of viruses.

Full virus name	Abbreviation	# Tests	Test Prop.	Neg.	Neg. in %	Pos.	Pos. in %	Pos. Prop.
Influenza virus A(H3N2)	FLUA(H3N2)	167904	3.83	151157	90.03	16747	9.97	2.79
Influenza virus A(H1N1)	FLUA(H1N1)	125326	2.86	118808	94.8	6518	5.2	1.09
Influenza Virus B	FLUB	326163	7.45	300287	92.07	25876	7.93	4.32
Human Parainfluenza Virus 1	HPIV-1	123871	2.83	122525	98.91	1346	1.09	0.22
Human Parainfluenza Virus 2	HPIV-2	123245	2.81	121999	98.99	1246	1.01	0.21
Human Parainfluenza Virus 3	HPIV-3	125028	2.85	119318	95.43	5710	4.57	0.95
Human Parainfluenza Virus 4	HPIV-4	80708	1.84	79530	98.54	1178	1.46	0.2
Human Metapneumovirus	HMPV	175581	4.01	168199	95.8	7382	4.2	1.23
Syncytial Respiratory Virus	RSV	246242	5.62	219994	89.34	26248	10.66	4.38
Rhinovirus	RV	152944	3.49	127580	83.42	25364	16.58	4.23
Enterovirus	EV	123017	2.81	117997	95.92	5020	4.08	0.84
Human Adenovirus	HAdV	156744	3.58	147823	94.31	8921	5.69	1.49
Human Coronavirus OC43	HCoV-OC43	106373	2.43	102841	96.68	3532	3.32	0.59
Human Coronavirus 229E	HCoV-229E	96859	2.21	95330	98.42	1529	1.58	0.26
Human Coronavirus NL63	HCoV-NL63	105063	2.4	103016	98.05	2047	1.95	0.34
Human Coronavirus HKU1	HCoV-HKU1	39190	0.89	38693	98.73	497	1.27	0.08
Human Bocavirus	HBoV	126788	2.89	121891	96.14	4897	3.86	0.82
Severe acute respiratory syndrome coronavirus 2	SARS-CoV-2	1661188	37.92	1253762	75.47	407426	24.53	67.95

2 Long-term trends

The following figures show the long-term absolute and relative infection counts for all viruses, in analogy to Figure 1 of the main paper.

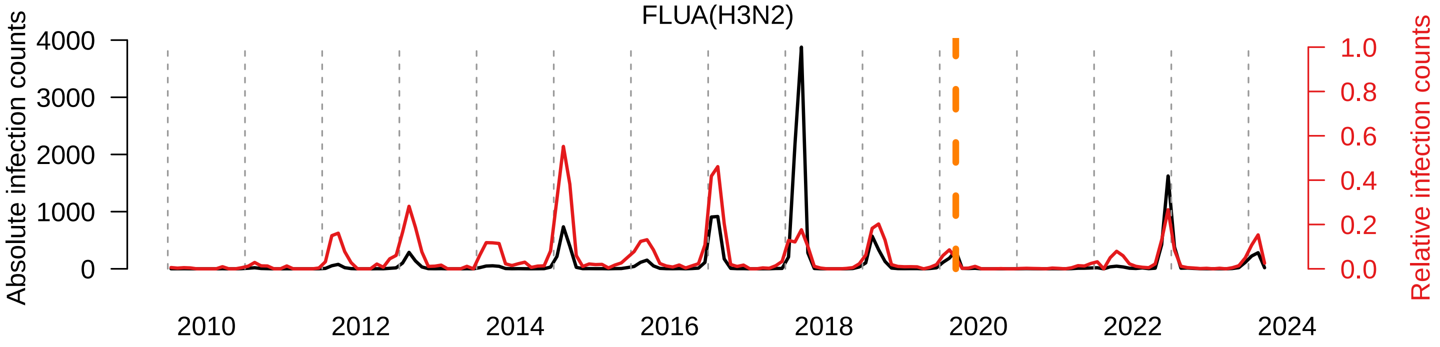

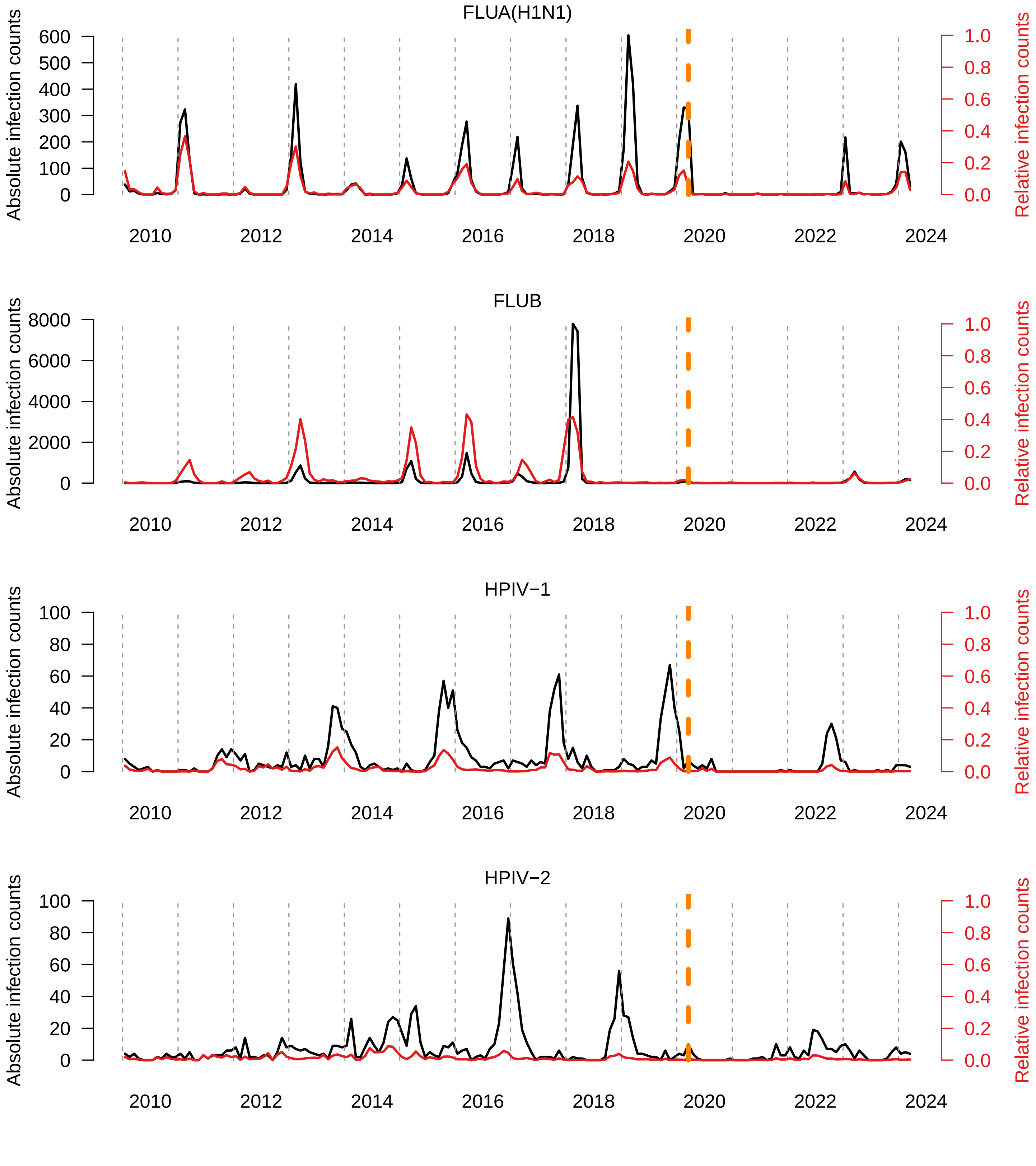
FLUA(H1N1)
FLUB
HPIV−1
HPIV−2
Absolute infection counts
Relative infection counts
2010
2012
2014
2016
2018
2020
2022
2024

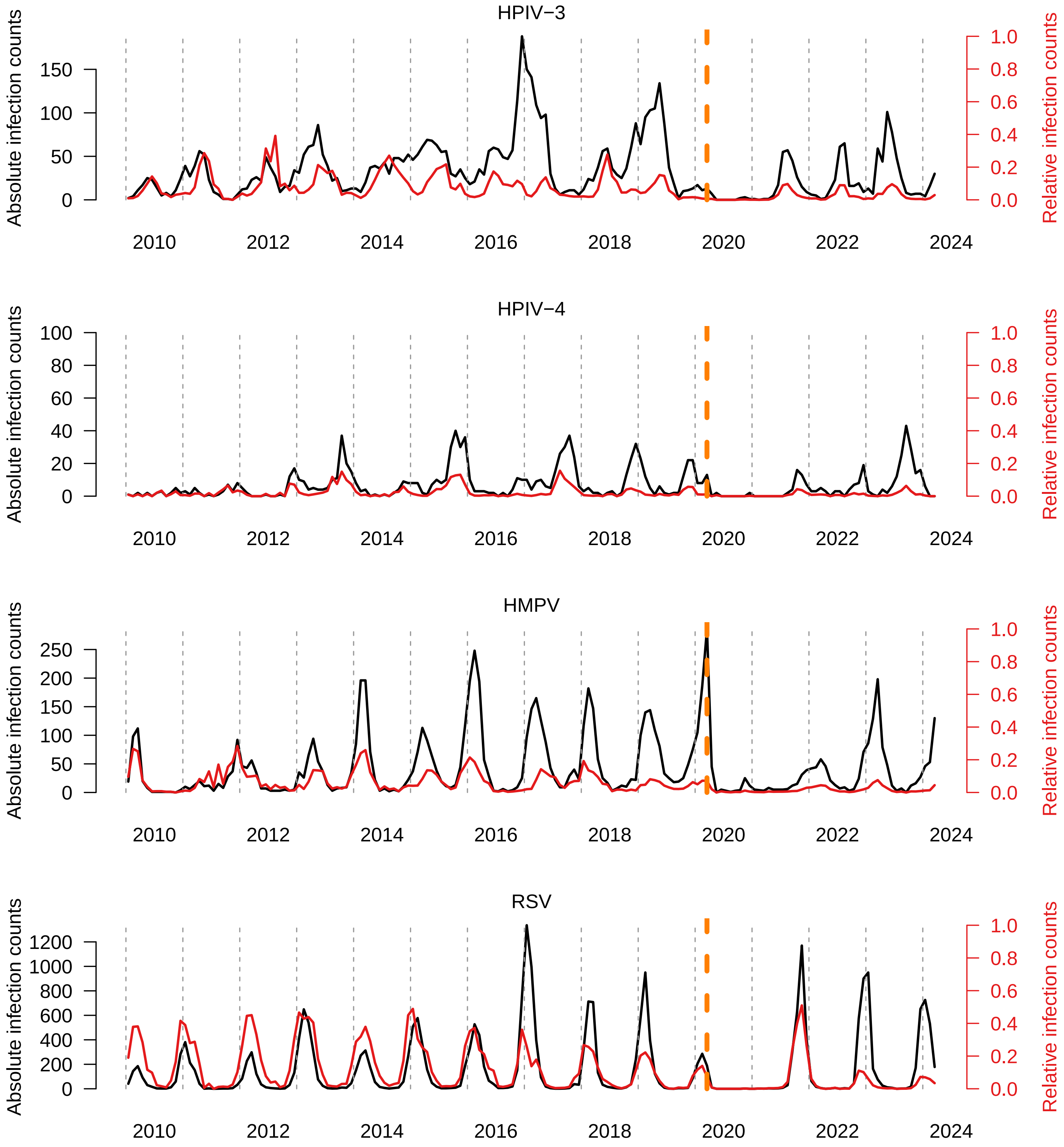
HPIV−3
HPIV−4
HMPV
RSV
Absolute infection counts
Relative infection counts
2010
2012
2014
2016
2018
2020
2022
2024
0
50
100
150
20
40
60
80
200
250
400
600
800
1000
1200
0.0
0.2
0.4
0.6
0.8
1.0

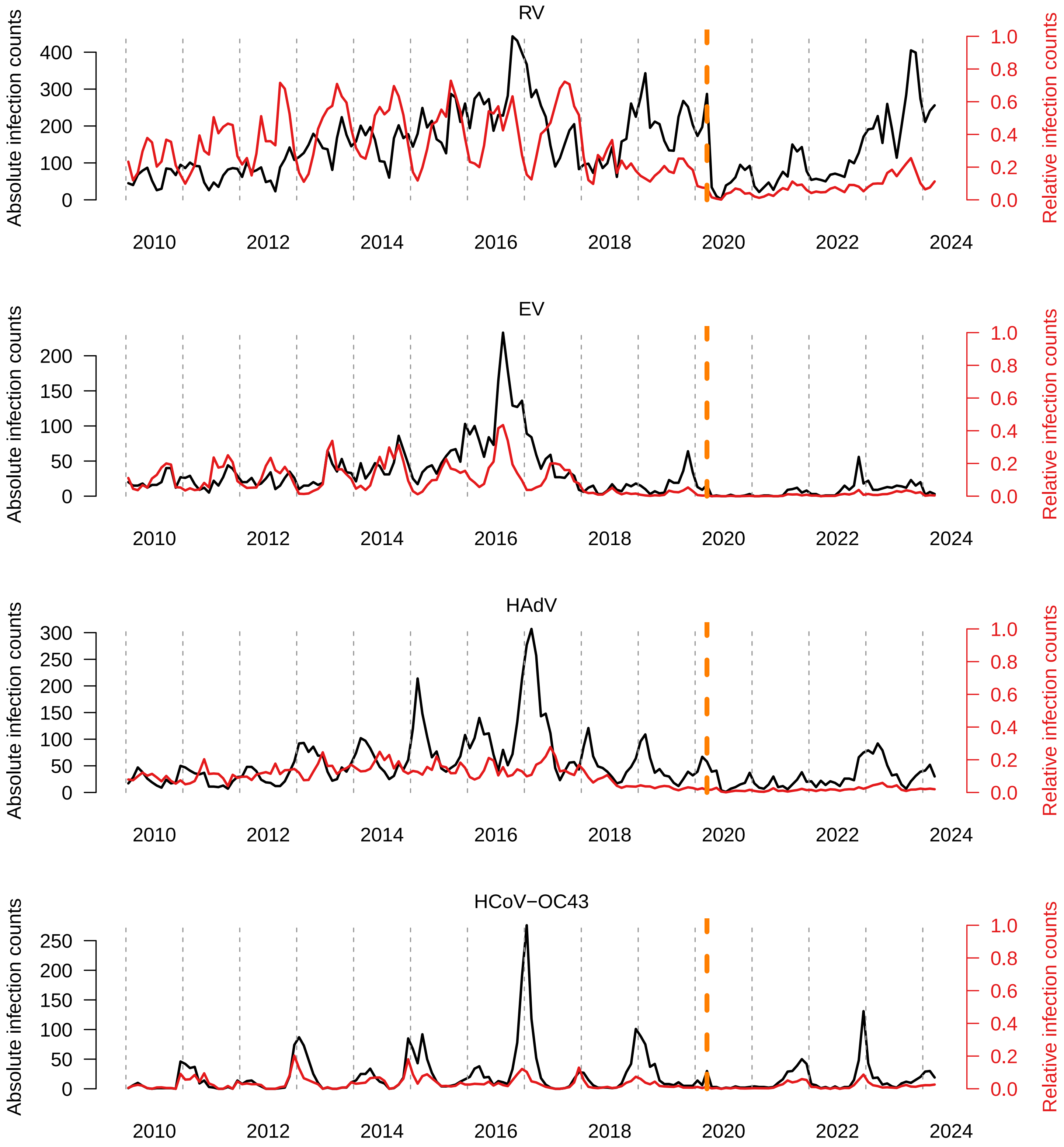
RV
EV
HAdV
HCoV−OC43
Absolute infection counts
Relative infection counts
0
100
200
300
400
50
150
250
0.0
0.2
0.4
0.6
0.8
1.0
2010
2012
2014
2016
2018
2020
2022
2024

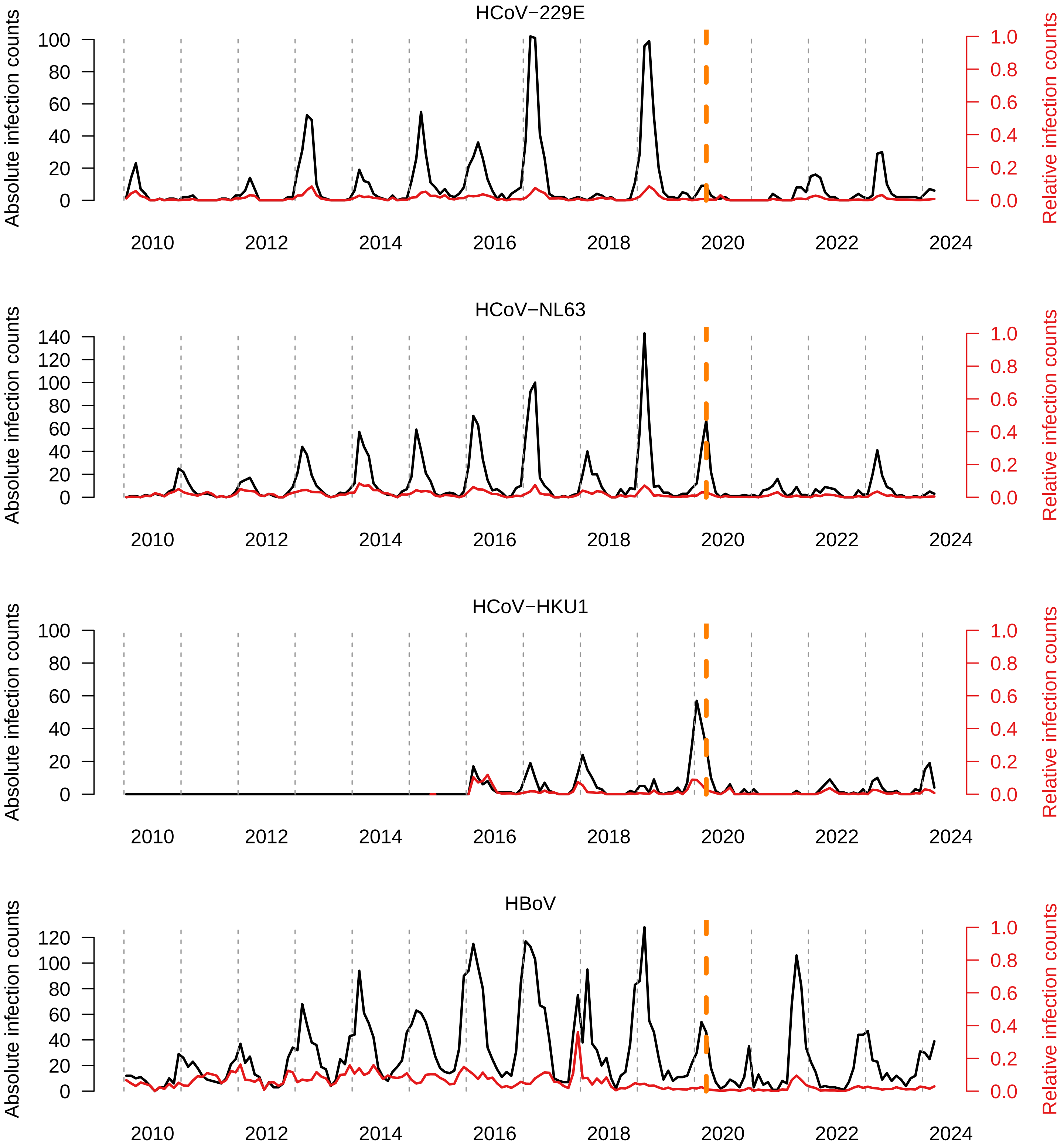

3 Smoothed infection counts & seasonality disruption quantification

The following figures show the smoothed infection counts and disruption statistics for all viruses in analogy to Figure 2A of the main paper.

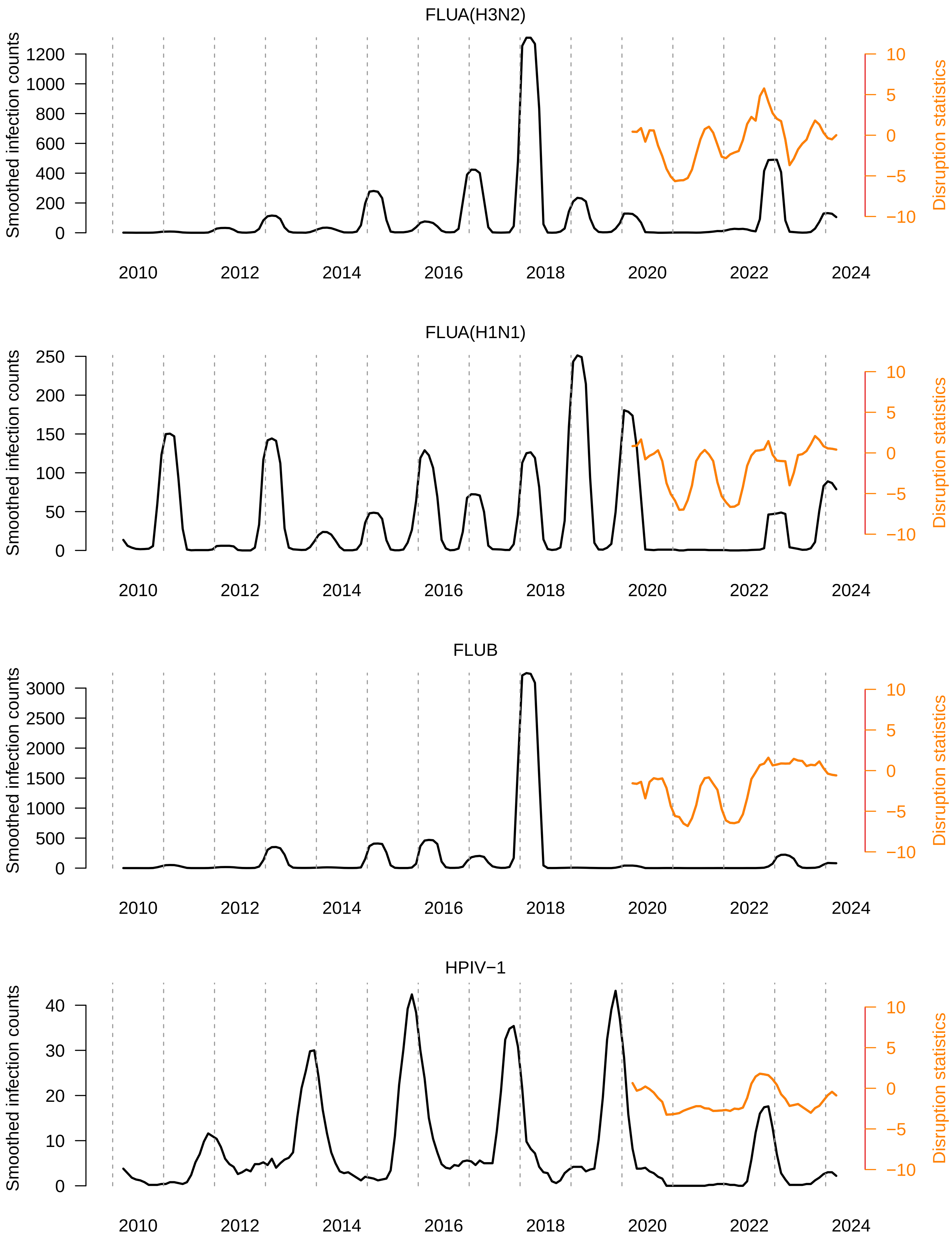
FLUA(H3N2)
Smoothed infection counts
0
200
400
600
800
1000
1200
Disruption statistics
−10
−5
0
5
10
2010
2012
2014
2016
2018
2020
2022
2024
FLUA(H1N1)
Smoothed infection counts
0
50
100
150
200
250
Disruption statistics
−10
−5
0
5
10
2010
2012
2014
2016
2018
2020
2022
2024
FLUB
Smoothed infection counts
0
500
1000
1500
2000
2500
3000
Disruption statistics
−10
−5
0
5
10
2010
2012
2014
2016
2018
2020
2022
2024
HPIV−1
Smoothed infection counts
0
10
20
30
40
Disruption statistics
−10
−5
0
5
10
2010
2012
2014
2016
2018
2020
2022
2024

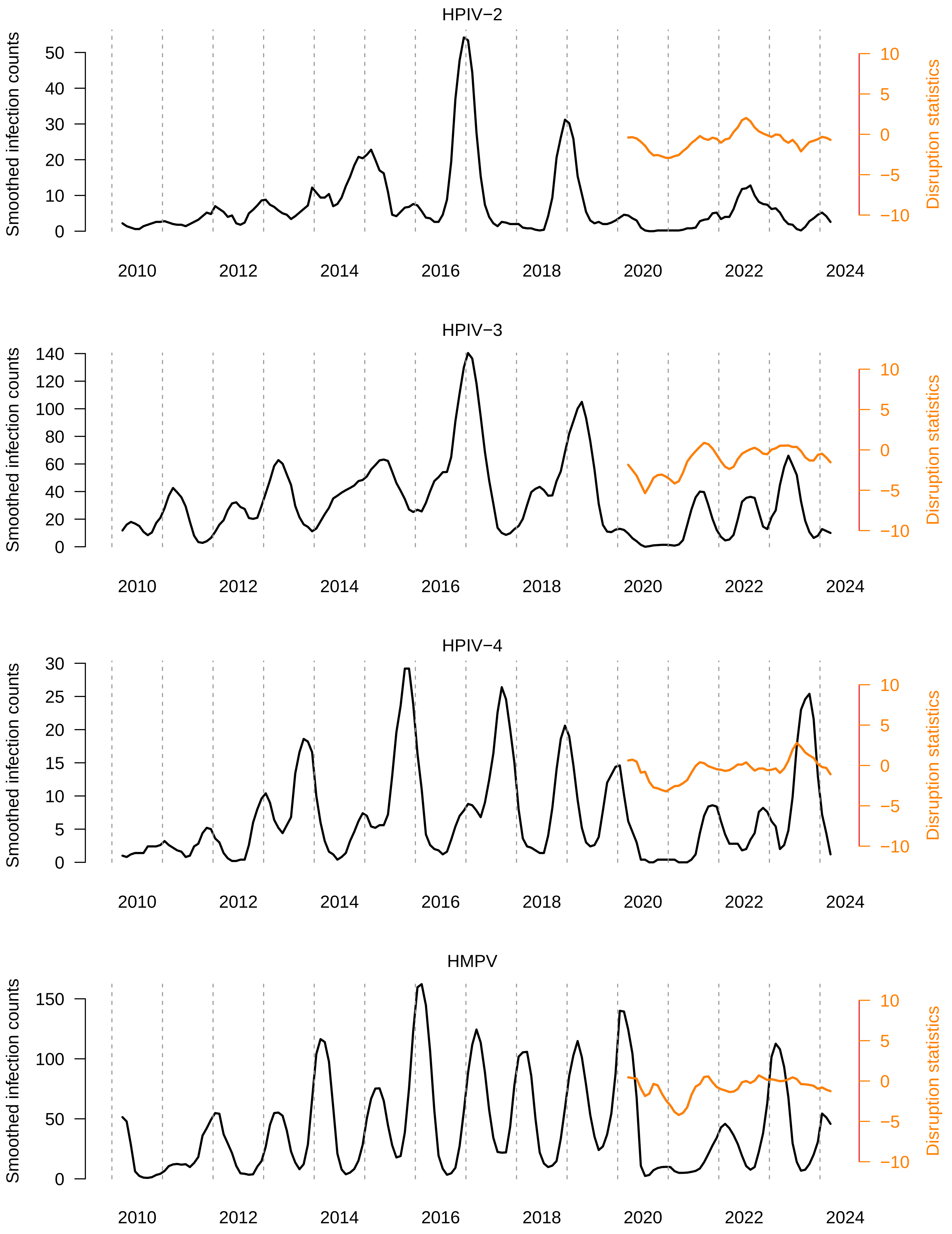
HPIV−2
HPIV−3
HPIV−4
HMPV
Smoothed infection counts
Disruption statistics
2010
2012
2014
2016
2018
2020
2022
2024

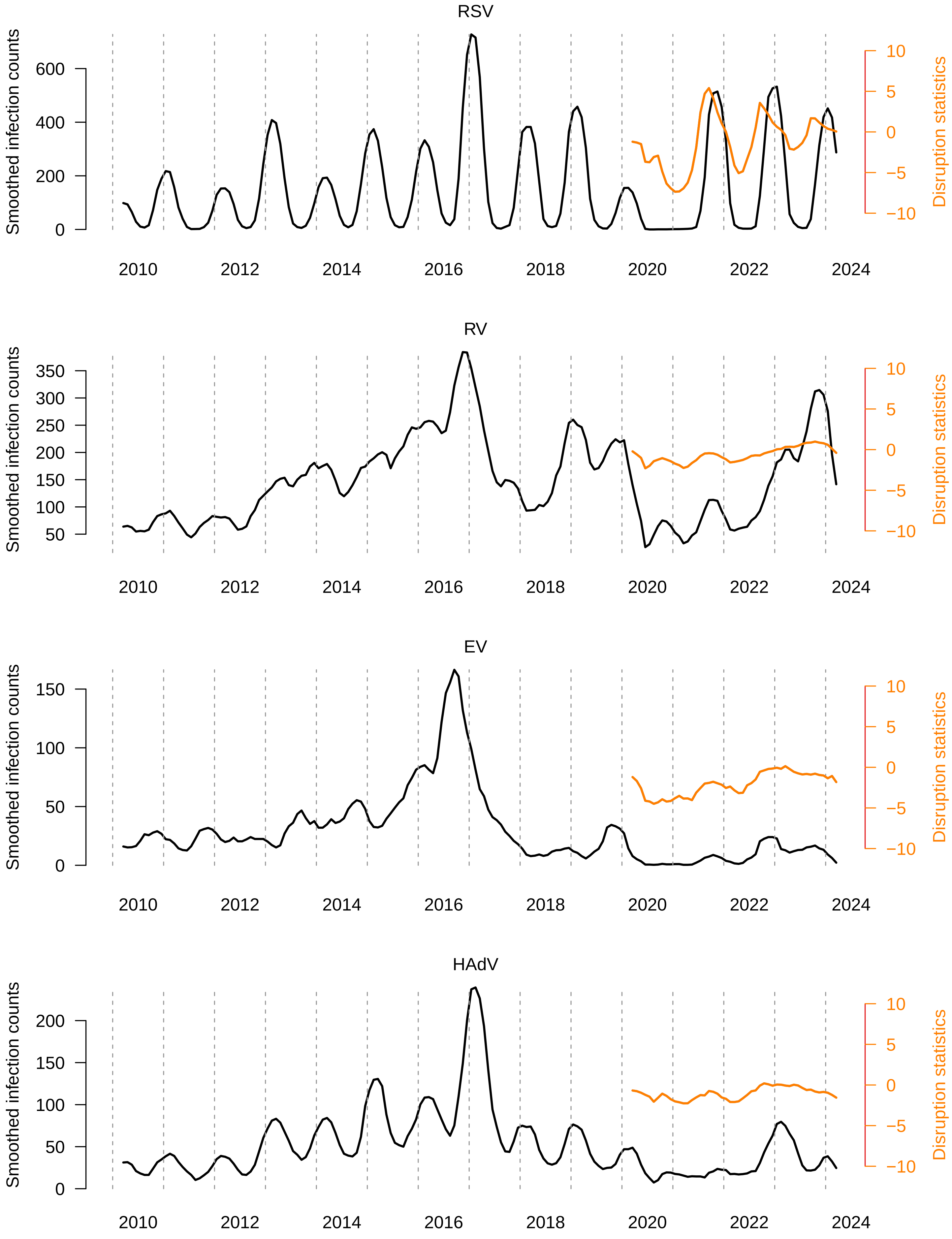
RSV
Smoothed infection counts
0
200
400
600
Disruption statistics
10
5
0
−5
−10
2010
2012
2014
2016
2018
2020
2022
2024
RV
Smoothed infection counts
50
100
150
200
250
300
350
Disruption statistics
10
5
0
−5
−10
2010
2012
2014
2016
2018
2020
2022
2024
EV
Smoothed infection counts
0
50
100
150
Disruption statistics
10
5
0
−5
−10
2010
2012
2014
2016
2018
2020
2022
2024
HAdV
Smoothed infection counts
0
50
100
150
200
Disruption statistics
10
5
0
−5
−10
2010
2012
2014
2016
2018
2020
2022
2024

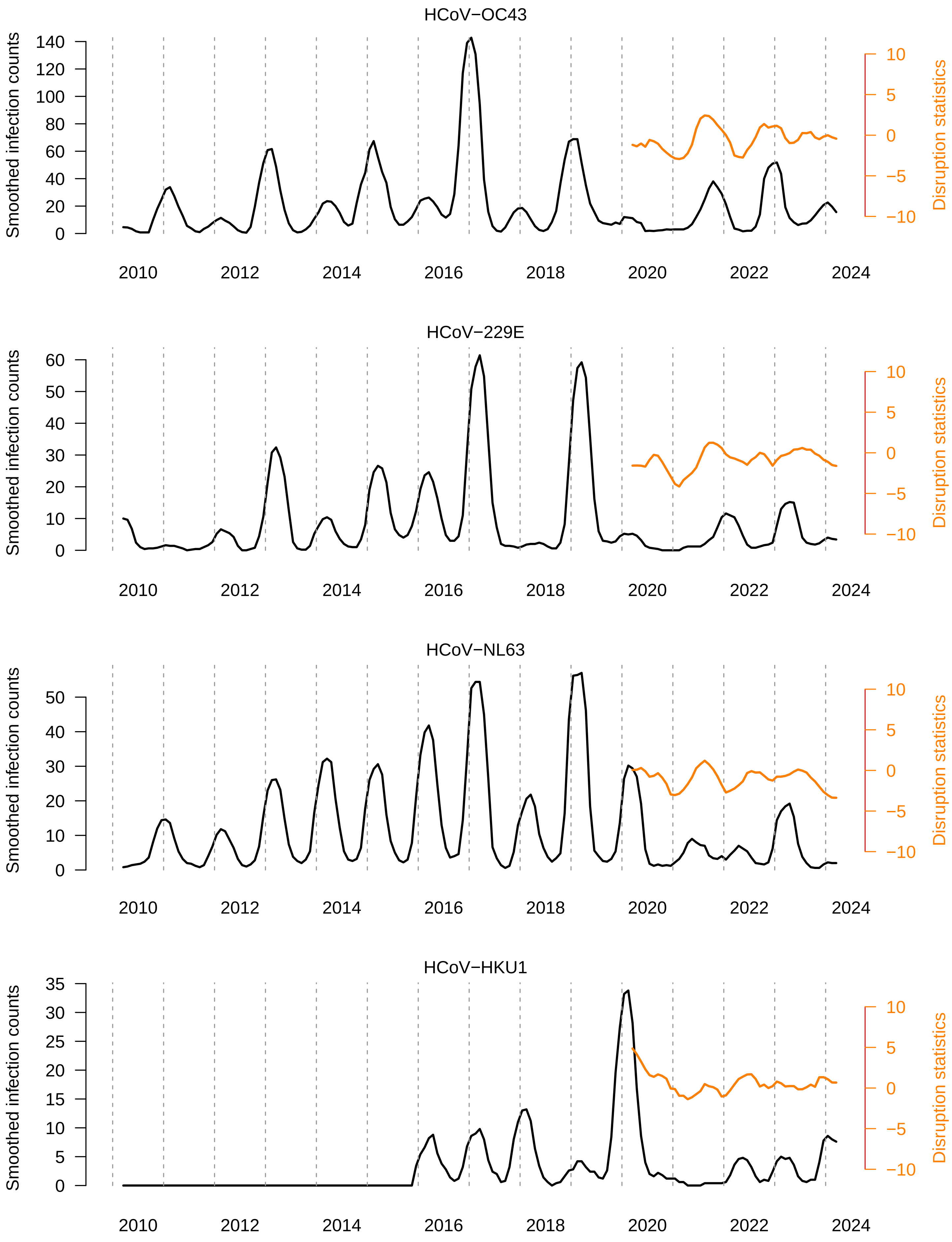
HCoV−OC43
Smoothed infection counts
Disruption statistics
HCoV−229E
HCoV−NL63
HCoV−HKU1
2010
2012
2014
2016
2018
2020
2022
2024

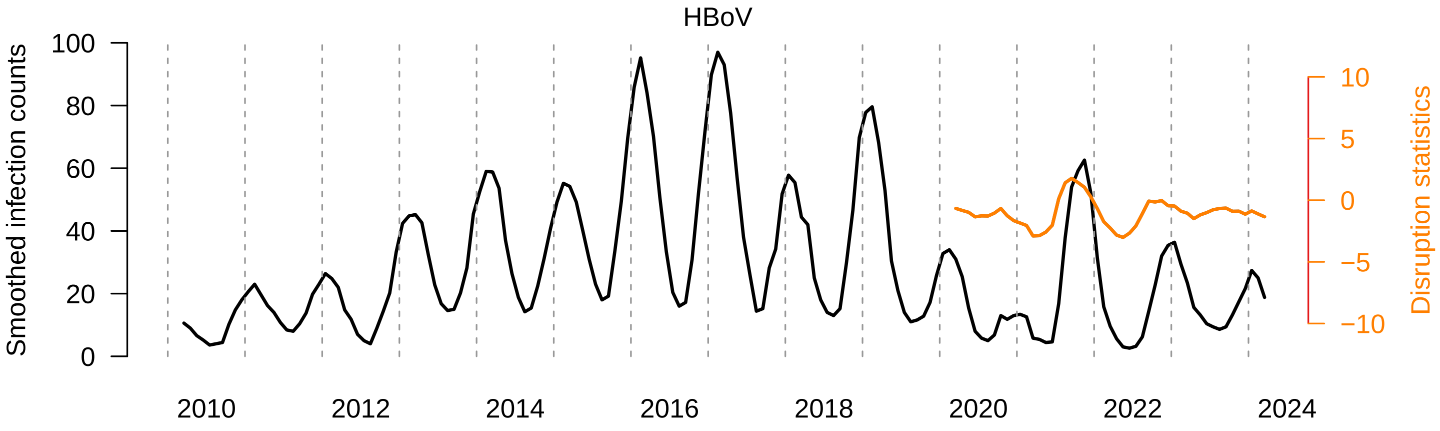

The following plot compares the SDI in the two years before the pandemic (March 2018–Febrary 2020) with that calculated after pandemic start (main paper):

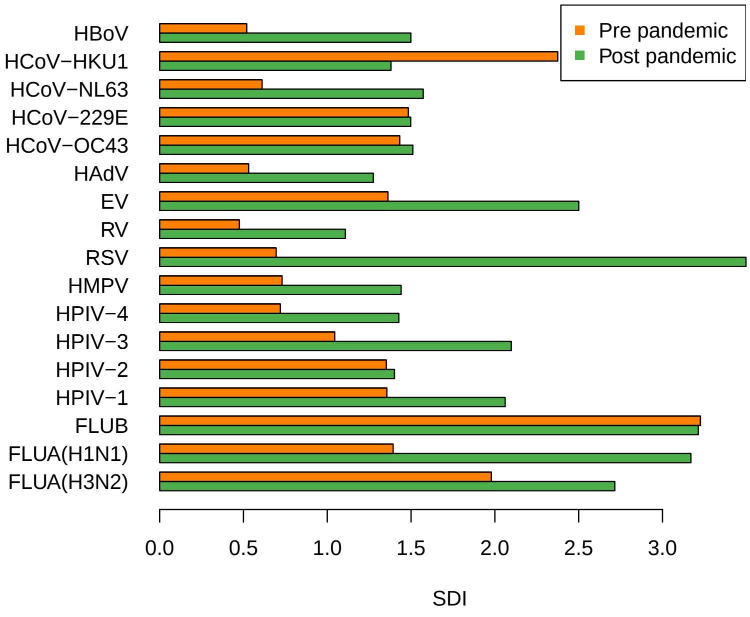

4 Infection counts vs NPIs

The following plots show an overlay between infection counts and Oxford stringency index for each virus, similar to Figure 3A of the main paper.

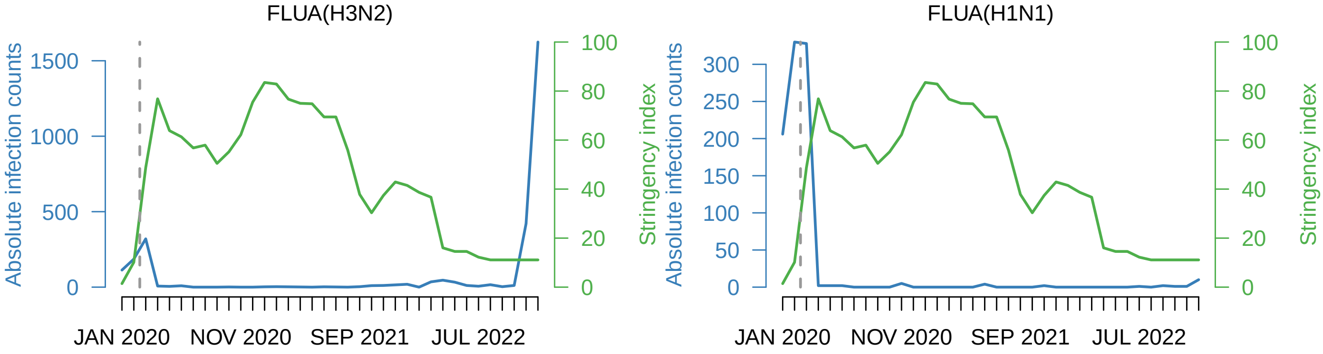

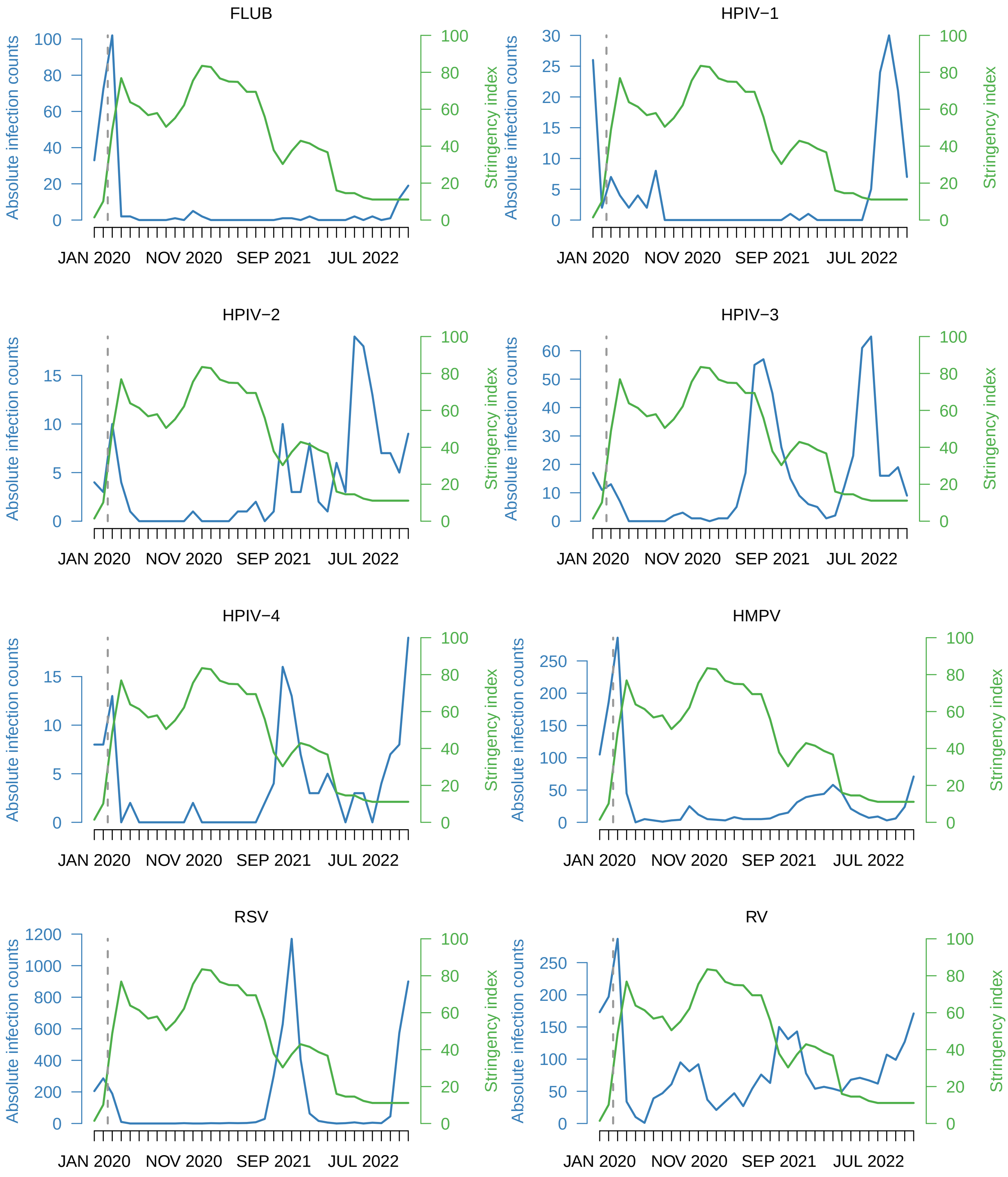
FLUB
HPIV−1
HPIV−2
HPIV−3
HPIV−4
HMPV
RSV
RV
Absolute infection counts
Stringency index
JAN 2020
NOV 2020
SEP 2021
JUL 2022
0
20
40
60
80
100
5
10
15
25
30
50
150
200
250
200
400
600
800
1000
1200

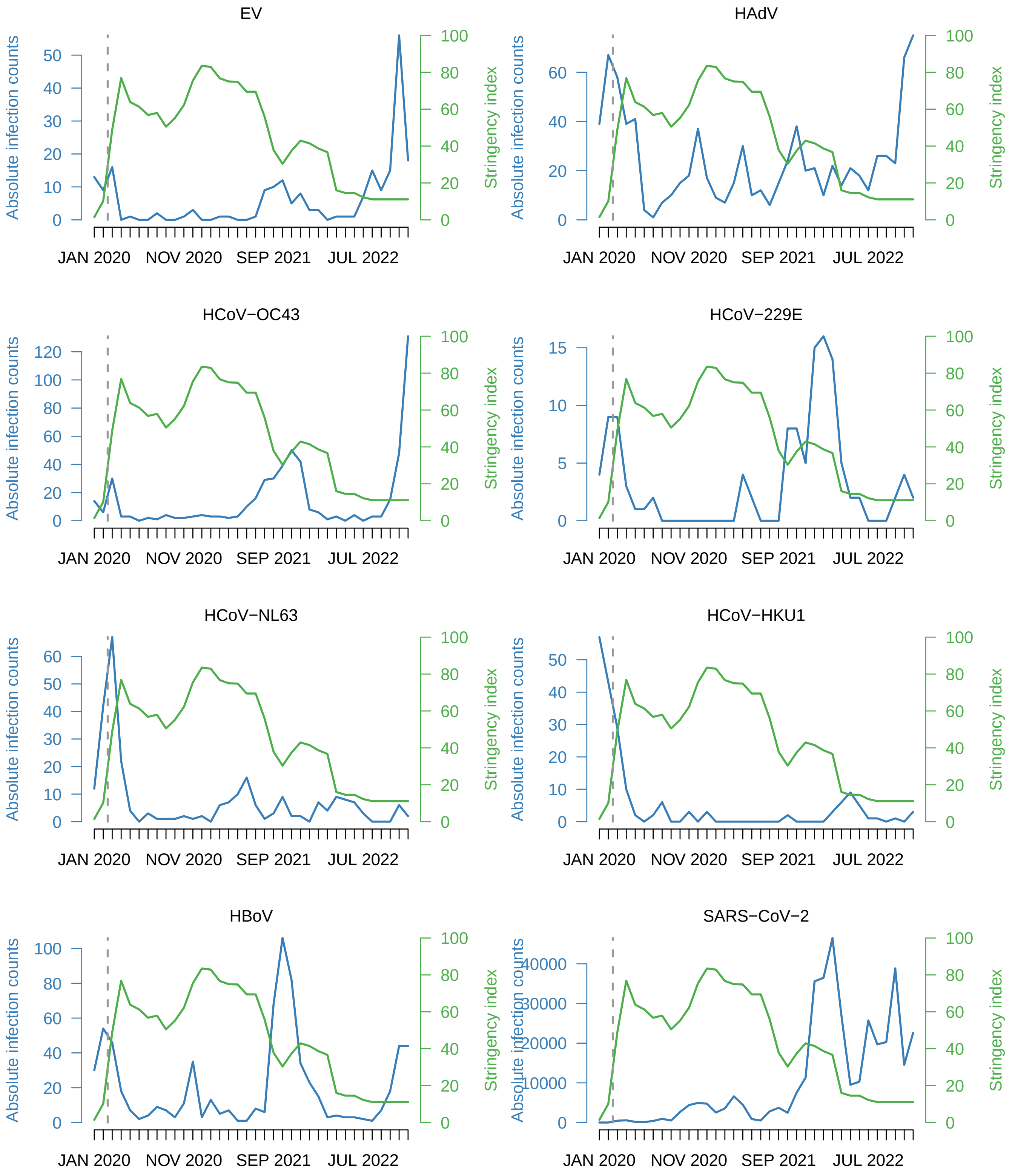

5 Correlation of NPIs with infection counts and disruption statistics

The following plots show the correlation between absolute infection counts and NPIs, both linear (Pearson) and rank (Spearman) correlation.

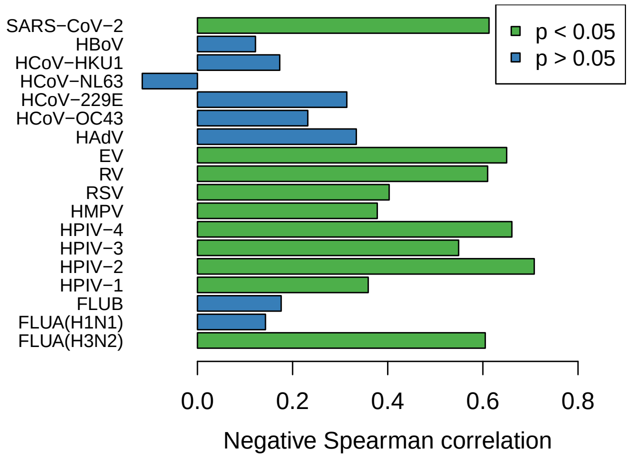

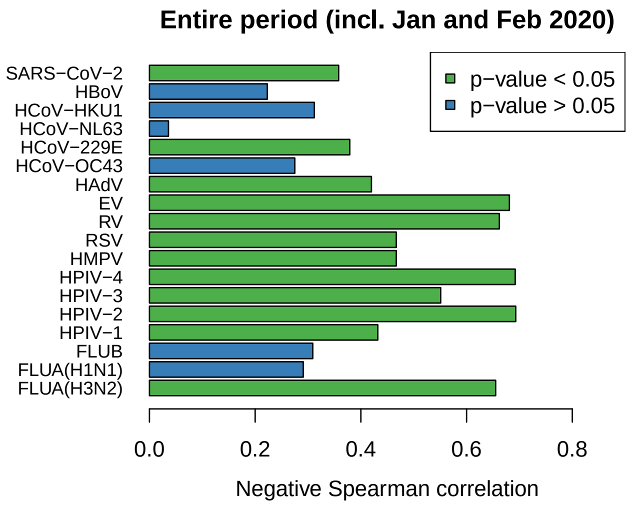

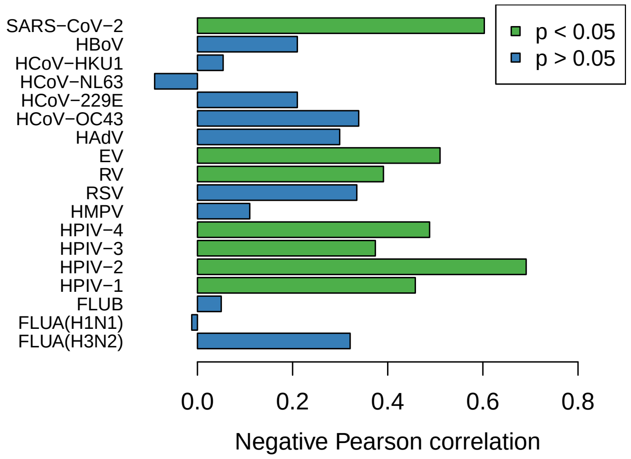

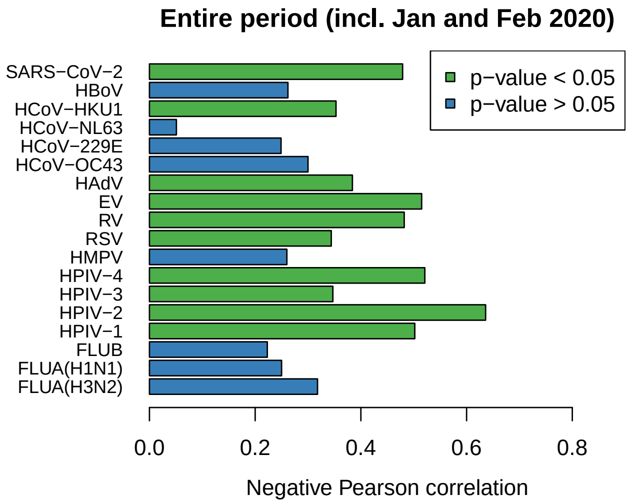

The following plots show the correlation between relative infection counts and NPIs, both linear (Pearson) and rank (Spearman) correlation.

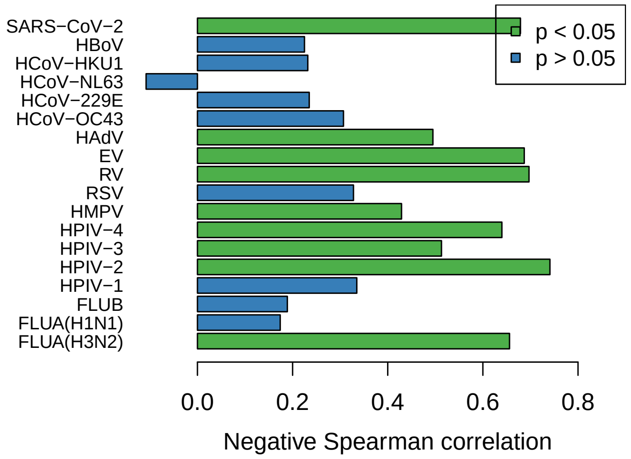

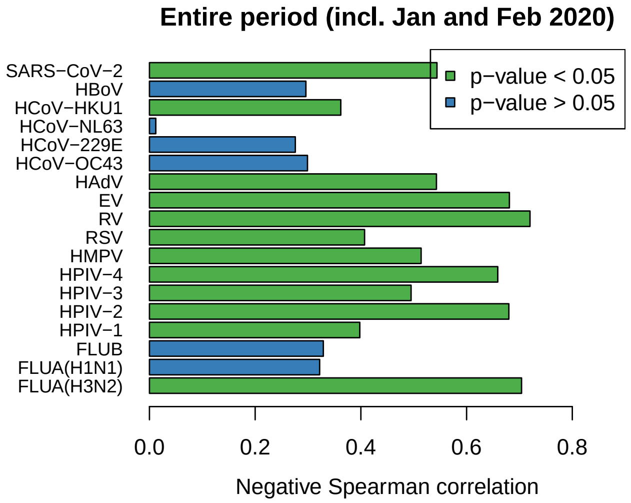

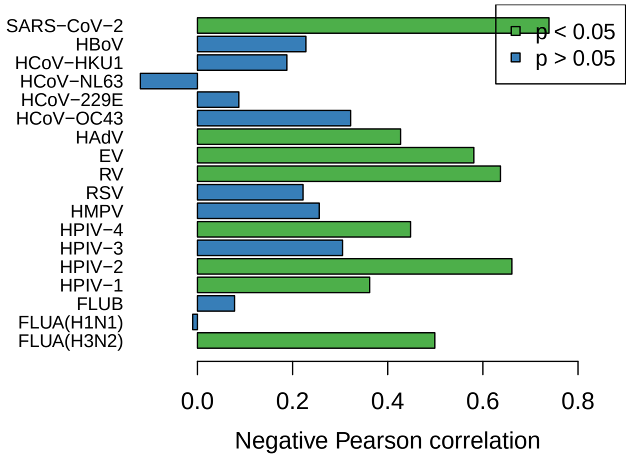

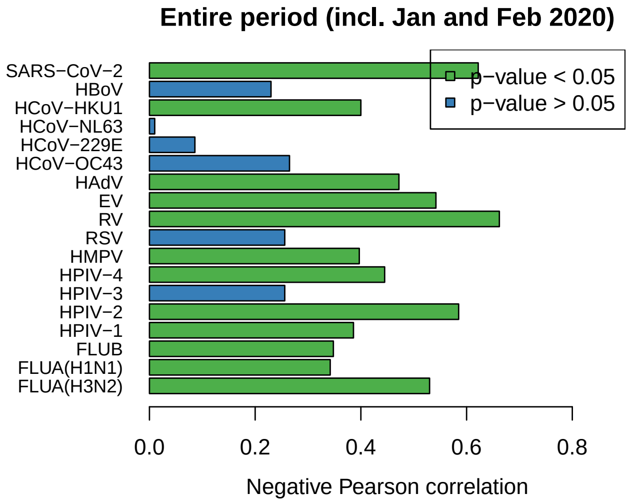

The following plots show the correlation between the disruption statistics and NPIs in the time frame March 2020–December 2022.

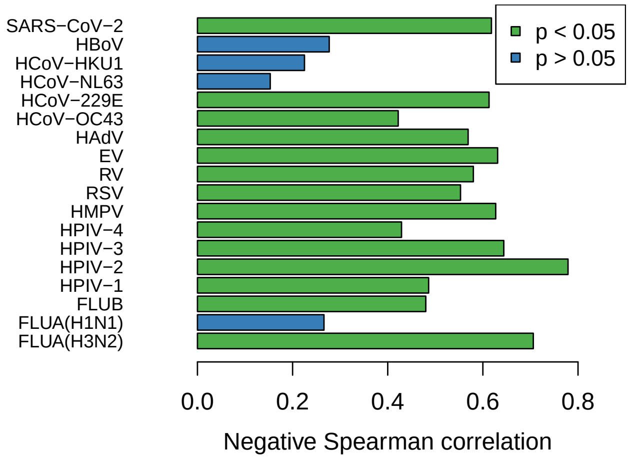

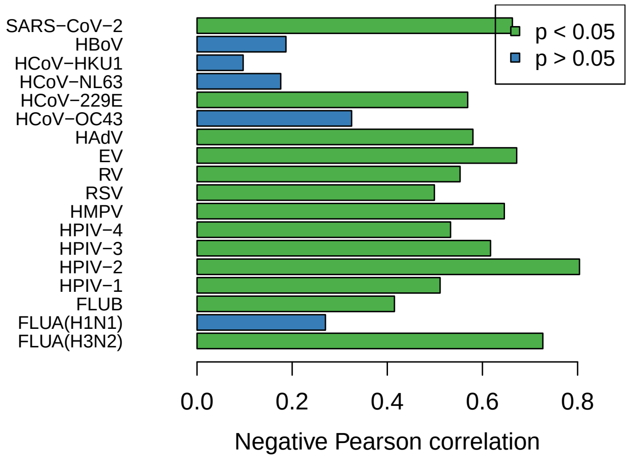

Supplement: Supplementary file 1 — Supplementary Material 1. [file 12889_2025_23983_MOESM1_ESM.pdf]
